# The Reach, Effectiveness, Adoption, Implementation, and Maintenance of Digital Mental Health Interventions for College Students: A Systematic Review

**DOI:** 10.1007/s11920-024-01545-w

**Published:** 2024-10-11

**Authors:** Madison E. Taylor, Michelle Liu, Sara Abelson, Daniel Eisenberg, Sarah K. Lipson, Stephen M. Schueller

**Affiliations:** 1 Department of Psychological Science, University of California, 214 Pereira Dr, Irvine, CA 92617 USA; 2 Department of Urban Health and Population Science, Lewis Katz School of Medicine, Temple University, Philadelphia, PA USA; 3 Department of Health Policy and Management, Fielding School of Public Health, University of California at Los Angeles, Los Angeles, CA USA; 4 Department of Health Law, Policy, and Management, School of Public Health, Boston University, Boston, MA USA

**Keywords:** College students, Mhealth, Universities, Digital mental health, Systematic review, Implementation science

## Abstract

**Purpose of Review:**

We evaluated the impact of digital mental health interventions (DMHIs) for college students. We organized findings using the RE-AIM framework to include reach, effectiveness, adoption, implementation, and maintenance.

**Recent Findings:**

We conducted a systematic literature review of recent findings from 2019–2024. Our search identified 2,701 articles, of which 95 met inclusion criteria. In the reach domain, student samples were overwhelmingly female and White. In the effectiveness domain, over 80% of DMHIs were effective or partially effective at reducing their primary outcome. In the adoption domain, studies reported modest uptake for DMHIs. In the implementation and maintenance domains, studies reported high adherence rates to DMHI content. While recruitment methods were commonly reported, adaptations and costs of implementation and maintenance were rarely reported.

**Summary:**

DMHIs for college students are effective for many psychological outcomes. Future work should address diversifying samples and considering implementation in a variety of college settings.

**Supplementary Information:**

The online version contains supplementary material available at 10.1007/s11920-024-01545-w.

## Introduction

Addressing student mental health is a major concern on college campuses. In 2023, the American College Health Association found that 45.9% of undergraduate students reported a history of at least one mental disorder diagnosis [1]. Additionally, many students experience considerable psychological distress (23.4%) and loneliness (53.3%) [1]. While college counseling centers have improved many aspects of treatment delivery (e.g., wait times and hours of operation) to meet the demand for mental health services [2], colleges are still searching for other novel solutions. Options include contracting with third-party vendors for additional after-hours care, improving off-campus referral networks, investing in prevention and early intervention, and using digital mental health interventions (DMHIs) [3].

DMHIs are designed to teach and deliver skills to improve mental health and well-being through mobile apps, web-based programs, virtual reality (VR), wearable devices, and/or video games [4]. Although some DMHIs may include human support, either by a peer, paraprofessional, or professional, we use the term DMHI to disambiguate from the use of technology only to connect a licensed mental health provider with a client (i.e., teletherapy). DMHIs have potential to be a useful treatment option on college campuses. The asynchronous format and self-guided nature of many DMHIs can provide increased flexibility for students with hectic schedules, who see lack of time as a barrier to treatment [5]. Likewise, DMHIs may be easily deployed and scaled across college campuses [3], as students receive many resources online. Meta-analyses of randomized control trials (RCTs) of DMHIs among college students have found them to be effective with small to moderate effect sizes (*d* = 0.52) [6].

Despite evidence supporting the effectiveness of DMHIs for college students, several open questions remain. A seminal review conducted by Lattie and colleagues [7••] examined the effectiveness, usability, acceptability, uptake, and adoption of DMHIs on college campuses across 89 studies. They found promising evidence of effectiveness, with 80% of the studies indicating that DMHI were effective or partially effective at reducing anxiety and/or depression in college student populations [7••]. However, only half of the studies assessed factors crucial for user engagement (e.g., acceptability and usability) [7••]. Even fewer studies explored factors related to implementing and maintaining DMHIs on campuses [7••]. These findings highlighted a need to expand research from considering only effectiveness towards evaluating how to best engage, implement, and sustain DMHI use among college students [7••].

Since these reviews were published [6, 7••], the mental health landscape on college campuses has changed considerably. The COVID-19 pandemic led to unprecedented disruptions and a notable increase in student mental health concerns [8]. Simultaneously, campus closures led to a greater interest in DMHIs from administrators [8]. The George Floyd protests and increases in Anti-Asian hate crimes in 2020 also drew additional attention to the mental health needs of BIPOC students [9, 10].

The aim of this study was to provide an overview of recent insights in the field by conducting a systematic review of the literature on DMHI for college students. Given the impact of the COVID-19 pandemic and presence of other reviews, we focused our review on the period after the Lattie and colleagues [7••] review, i.e., 2019 to 2024. We organized our findings within the RE-AIM framework to better identify gaps in our knowledge that may inhibit the dissemination and implementation of DMHI on college campuses. Finally, we provide suggestions for how DMHI research for college students should expand going forward.

## Methods

### Eligibility Criteria

Studies were eligible for inclusion in the review if they were empirical studies conducted between 2019–2024 that (1) reported results on DMHIs and (2) had college or other post-secondary education students (e.g., professional and trade) as the target population. DMHIs were defined as (1) having a technology-based intervention component (e.g., web-based platforms, apps, VR) and (2) delivering these intervention components to prevent and/or treat mental health conditions or increase well-being [11]. Studies were ineligible if they were not written in English and had no English translation, their results were not DMHI-related, the target population was unclear, or they did not present empirical data.

### Database Search Strategy and Selection

To cover a variety of psychological and technology disciplines, the literature search used the PsycINFO, PubMed, and ACM Guide to Computer Learning databases. A comprehensive search strategy was developed using keywords to describe college/university students, mental health conditions (i.e., depression, anxiety, stress), technology, and digital mental health interventions (see Supplement 1 for a full list of keywords). Each database was searched from 2019 to 2024. This yielded a total of 2,701 articles. Research assistants did an initial screening of article abstracts according to eligibility criteria. The first two authors (MET and ML) re-assessed the titles and abstracts for eligibility criteria. Following title and abstract screening, MET and ML independently reviewed the full-text of all articles to identify those that met inclusion criteria. Any discrepancies were identified and resolved by discussion until a consensus was reached.

### Data Synthesis

**Fig. 1:**
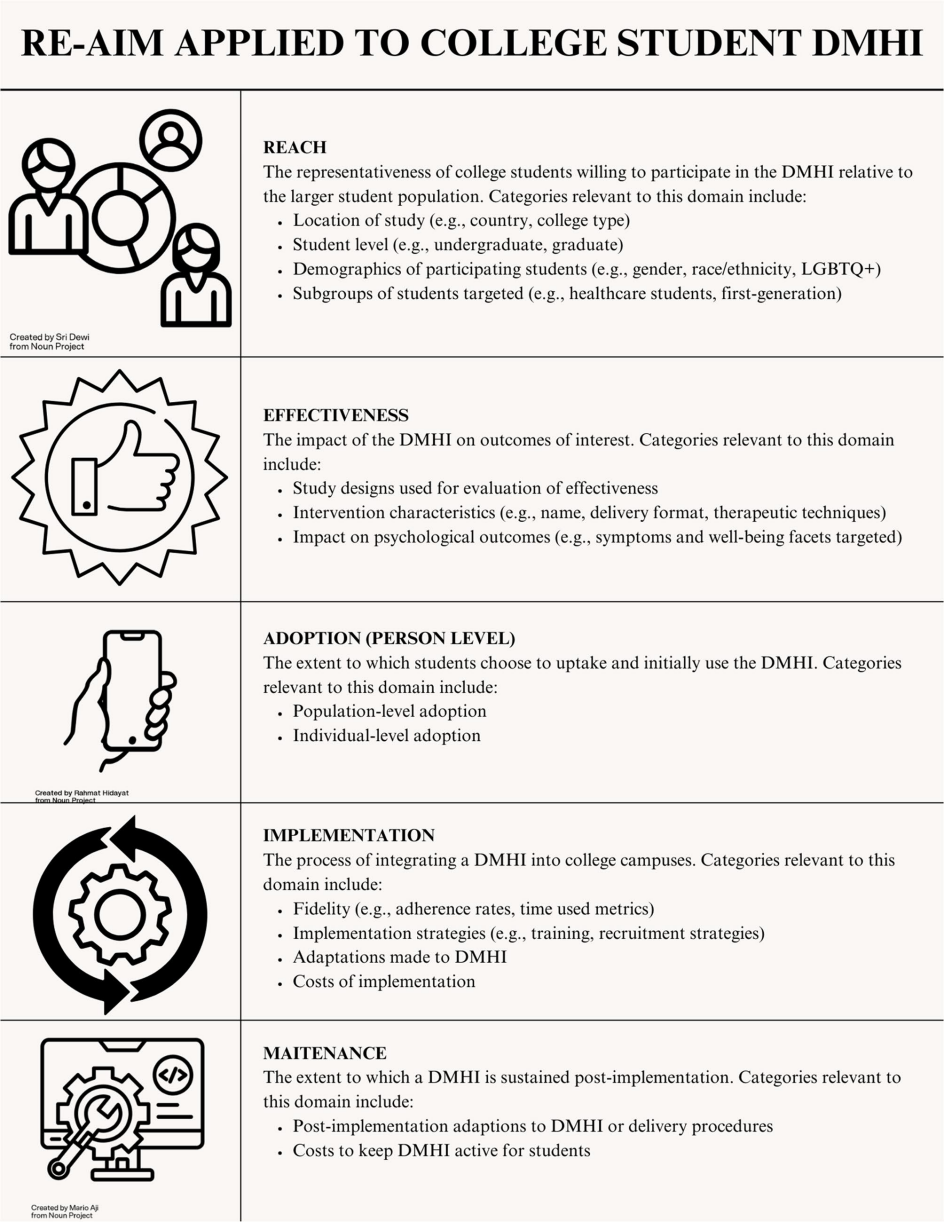
Chart of RE-AIM domains and their application to DMHIs for college students. Note. “Demographics” icon by Sri Dewi, “Holding the Phone” icon by Rahmat Hidayat, and “Tools” icon by Mario Aji from thenounproject.com CC BY 3.0

The content of eligible articles was mapped onto the RE-AIM framework [12••]. The first author (MET) developed a coding scheme based on the types of content that fall under each domain of RE-AIM (see Fig. 1). A notes category was used to code any aspects of the study found relevant but that did not fit neatly into the existing coding categories.

#### Reach

Reach refers to the percentage and characteristics of those who receive an intervention, especially with respect to the target population [12••]. For this domain, eligible studies had the following information coded: the country where the study took place, the level of a college student (i.e., undergraduate, graduate, professional), college type (e.g., university, four-year liberal arts, community college), and sample demographics (e.g., gender/sexuality and race/ethnicity). We coded studies if they had majority (> = 60%) female participants, majority (> = 60%) male participants, or roughly equal female and male participants. For studies taking place in the United States and Canada, we also coded if the rates of race/ethnicity of participants matched the total enrollment rate for students of different race/ethnicities across college campuses [13].

#### Effectiveness

Effectiveness refers to the impact of the program on target outcomes [12••]. In the case of DMHIs, this includes reductions in or prevention of mental disorder symptoms or improvement in well-being [12••]. In the effectiveness domain, we coded the name of the DMHI used, primary and secondary outcomes, and the extent to which DMHI was successful in changing the outcomes of interest. We also coded the technology’s delivery format, the therapeutic techniques used in the intervention, and the study’s design, as these intervention and study characteristics provide further context to effectiveness findings.

#### Adoption

Adoption refers to the proportion of people who are willing to initiate a program [12••]. For DMHIs, this is often conceptualized as uptake [12••]. Adoption can be considered from both a population- and individual-level. The population-level addresses what percent of students join a study among all those who are approached or recruited [14]. The individual-level is defined as the number of participants who, once enrolled in the study, download and use a DMHI at least once [15•]. We recorded the relevant population- and individual-level metrics for the eligible studies. This included doing additional computations if the relevant information was available but these metrics were not calculated in the original article (e.g., using participant flow charts from RCTs).

#### Implementation

Implementation refers to the integration of a program into the deployment setting, including whether it is used as intended [12••]. Although the use of an intervention as intended is often determined by provider fidelity to the intervention protocol, this concept aligns with user engagement metrics such as adherence and usage time for DMHI as many are self-guided or direct-to-consumer. Adherence is measured as completion of an expected level of content and alignsghts with fidelity [15•]. To represent adherence, we recorded rates of content completion in DMHIs. We use the term usage time to refer to metrics that quantify how long students spent using the DMHI. This can include time spent on the platform overall, time spent on specific sessions, or sustained use (i.e., “remaining active in using the intervention for some period of time after downloading”; [15•]). As such, we recorded whatever usage time metrics were reported by the studies. For determining implementation activities related to integration into campus settings, we coded the implementation site, implementation strategies used, any adaptations made to the DMHI for implementation, and costs associated with DMHI implementation.

#### Maintenance

Maintenance refers to the extent to which a program is sustained over time, including any implementation strategies or adaptations used to promote sustainment and maintenance costs [12••]. We coded maintenance strategies used, post-implementation adaptations, and costs associated with maintenance.

## Results

**Fig. 2:**
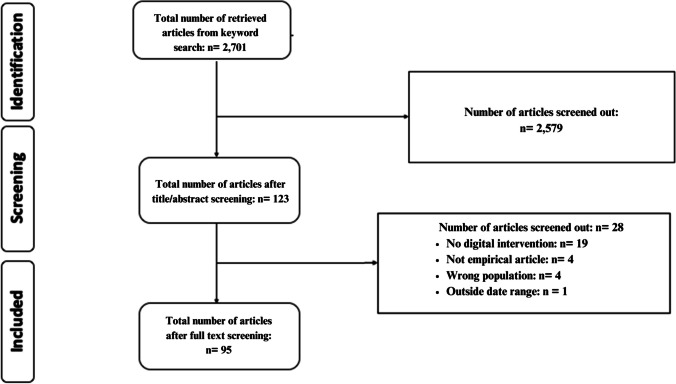
Preferred reporting items for systematic reviews and meta-analyses (PRISM) flow diagram

The search retrieved 2,701 articles that were screened at the title and abstract stage. The full text of 123 articles were reviewed for inclusion, resulting in 95 articles that met the inclusion criteria (See Fig. 2 for the flow diagram and Supplemental 2 for a table of all articles).

### Reach

Of the 95 studies, most 60.0% occurred in the United States or Canada (see Table 1). Undergraduate students were the population for 47.9% of the studies, 30.2% of the studies had a combination of graduate and undergraduate students, and 5.2% focused specifically on graduate students. Just over half of the studies (56.2%) took place at large or mid-sized universities. Only one study took place at a liberal arts college and two at community colleges.

**Table 1:** Sample demographics across studies

		N (Studies)	%
Study Location	US/Canada	57	60.0
	Other	38	40.0
College Level	Undergraduate	49	51.6
	Graduate/Professional	13	13.7
	Community College	1	1.1
	Combo	32	33.7
Gender Distribution	Female (greater or equal to 60%)	76	80.0
	Male (greater or equal to 60%)	3	3.2
	Equal distribution	12	12.6
	Not reported	4	4.2
LGBTQ + Reported*	Sexuality	12	12.6
	Non-Cisgender	28	29.5

*Note. N for this category will not add up to 95

Women were more likely to enroll in the studies with 80.0% of the studies reporting over 60% of the sample as female. Only 12.6% of the studies reported demographic data on participants’ sexuality; students who identify as non-heterosexual comprised 5%-35.9% of their samples. One study reported that 62.5% of the students in the sample identified as non-heterosexual, but it had a sample size of 8 participants [16]. 29.5% of studies reported demographic data on non-cisgender students.

For the 57 studies conducted in the United States and Canada, Hispanic/Latine students were represented at rates at or above their national enrollment rate in colleges (i.e., 16%) in 24% of studies [13]. Black/African American students were represented at rates at or above their national enrollment rate in colleges (i.e., 10%) in 29% of studies [13]. Asian students were represented at rates at or above their national enrollment rate in colleges (i.e., 6%) in 57% of studies [13]. American Indian and Alaskan Native students were represented at rates at or above their national enrollment rate in colleges (i.e., 0.6%) in 24% of studies [13]. Native Hawaiian or Other Pacific Islander students were represented at rates at or above their national enrollment rate in colleges (i.e., 0.2%) in 17% of studies [13].

Some studies targeted specific demographics of students during recruitment. Studies that specifically developed interventions for students in healthcare made up 6.3% of the studies surveyed [17–22]. Three other studies recruited specialized samples: students who experienced IPV [23], first-year students [24], and veteran students [25].

### Effectiveness

#### Study Designs

68 of the 95 studies evaluated the effectiveness of the DMHI on psychological outcomes. RCTs were 72% of these studies. Of the RCTs, 40.8% had a waitlist control, 38.8% had an active control, and 20.4% had multiple comparison conditions (e.g., treatment as usual and a waitlist). Pre-post designs were used in 24% of these studies and the final 2% of studies used other quasi-experimental designs. 32.4% of studies only recruited participants with mental health concerns, including those with clinically-elevated mental health symptoms or identified risk factors.

#### Intervention Characteristics

Few DMHIs were evaluated in multiple studies. The most commonly tested DMHIs were Studicare (6.0%), the ACT Online Program (4.0%), and Headspace (3.0%). Apps and other mobile platforms were the most common delivery format, used in 44.2% of studies. Another 38.2% of the DMHI in these studies used web-based delivery. Other delivery formats included online courses, videos, and ecological momentary interventions (EMIs). Of the DMHI tested, 5.9% had an in-person adjunct component (e.g., workshop).

The DMHI in these studies used a variety of therapeutic techniques and addressed many different mental health issues. CBT (36.7%), ACT (25.0%), and mindfulness (30.9%) were the most common therapeutic techniques.

#### Impact on Psychological Outcomes

The most common clinical outcomes assessed were depressive symptoms (50.0%), anxiety symptoms (44.1%), and stress (41.2%). Trait mindfulness (e.g., Freiburg Mindfulness Inventory; [26]) was also a common outcome measure, being featured in a quarter of studies (25%). Other outcome measures assessed include quality of life, flourishing, adjustment, student performance, well-being, sleep disturbance, experiential avoidance, eating disorder symptoms, self-efficacy, negative affect, and knowledge of mental health care.

DMHI were effective for the primary outcome(s) measured in 47% of the studies. An additional 34% studies found that their DMHI was partially effective (i.e., a significant change in the desired direction observed in some but not all of the primary outcome(s) measures or were effective for only a subset of students). Only 19% of studies found the DMHI had no effect (e.g., no desired change over time or greater change in DMHI compared to control) on the primary psychological outcome(s) measured. Of the 30 studies that had anxiety symptoms and/or diagnoses as a primary outcome measure, 60% found that the DMHI was effective at reducing the anxiety at the highest level of control offered. For the 23 studies that had depression symptoms and/or diagnoses as a primary outcome measure, 83% showed effectiveness in reducing depression symptoms at the highest level of control offered.

### Adoption

#### Population-Level

20 studies reported on adoption at the population level (see supplemental 2). Between 0.5% to 93.7% of students responded to study participation invitations in the 10 studies that had sufficient data to determine this metric (median = 13.9%). Between 3.1–91.4% of students consented to participate in study after the eligibility was determined in the 16 studies that reported this metric (median = 48.8%).

#### Individual-Level

16 studies reported adoption rates for the DMHI on the individual-level (see supplemental 2). For the 12 studies using “traditional” recruitment strategies (e.g., participant pools, study advertisements), uptake rates were generally high, ranging from 65% to 100% [17, 18, 27–29, 30•, 31–36]. Six out of 12 of these studies reported compensating participants monetarily or with course credit [28, 29, 31, 33, 35, 36]. Adoption rates were higher on average for the non-paid samples (M = 92%) than the paid samples (M = 77.6%). For the 4 studies that reported uptake for DMHIs implemented into college campuses, uptake varied more greatly. These rates ranged from 26.7% to 90.5% [37•, 38, 39•, 40].

### Implementation and Maintenance

#### Usage Time

See supplemental 2 for a full list of usage time metrics. For 3 studies using single-session intervention (SSI) DMHIs, students spent between 30–40 min on average completing the SSI [29, 41, 42•]. For apps, 3 studies that reported on session duration found that users spent 3-13 minutes on average per session [43–45]. Students who were given access to modular web-based treatments or apps over several weeks generally spent hours of time on their DMHI. Time spent ranged from 37.9 min [46] to 120 min [47] on average each week, and 3 h and 18 min [32] to 48 h [48] over the whole study duration. In terms of sustained use, studies generally found that students used their DMHI less towards the end of the multi-week period than at the beginning [49, 50]. One study reported consistently high use over a 6-month period and that study used an ecological momentary intervention (EMI) [36].

#### Adherence

Adherence rates were reported in 28 studies (see Supplemental 2), which were generally high. For 17 studies that reported the percentage of content completed, 71% had students complete over 50% of the DMHI's content on average and 53% had students complete over 70% of content available. For 13 studies that reported the percentage of participants that met a specific criterion of expected use, 77% had over half of participants meet their criterion.

#### Integration Factors

Of the 95 studies reviewed, only 11 studies reported on implementation activities (see supplemental 2). An additional 4 feasibility and effectiveness studies used DMHI that were already integrated into existing structures on college campuses. Three studies involved the implementation of DMHI across multiple universities [37•, 38, 51]. The most common recruitment strategy involved using university listservs to advertise the DMHI and educating staff and faculty on the availability of the DMHI. Two studies reported having an onsite coordinator or representative for the DMHI [38, 40].

Outside of recruitment strategies, studies largely did not report other implementation activities. Four studies reported on adaptation processes to modify the DMHI for use among the intended college student population. Wasil and colleagues [42•] made adaptations to the vignettes and activities in their pre-existing COMET SSI to make it more applicable to the challenges college students were facing during COVID-19. Chung and colleagues [51] replaced the American psychologist voice-over for mindfulness exercises with a British psychologist voice-over to suit the context of delivery in UK colleges. Benjet and colleagues [27] culturally adapted SilverCloud for Mexican and Colombian college students. Pankow and colleagues [50] adapted the symptom monitoring and care planning platform iSpero into a self-guided and therapy adjunct tool for students through biweekly meetings with community stakeholders, but did not describe specific adaptations made. Two studies reported on the potential costs of implementing and maintaining a DMHI [30•, 52•]. Gatto and colleagues [30•] reported that their DMHI BERT took a research team member 1-2 hours a day to manage data and send email reminders and additional materials. Davis and colleagues [52•] estimated that their DMHI ACT Online Guide would need 20 hours a week of support to conduct technical upkeep and continued recruitment efforts.

## Discussion

In recent years, many studies have evaluated DMHIs on college campuses. We found that a majority of these studies were conducted at four-year universities in the United States and Canada. Most studies recruited White and female students and few matched the demographics and diversity of college students nationally. The DMHIs evaluated had a wide range of delivery formats and incorporated skills from various treatment modalities. DMHIs appeared to be effective, with over 80% of studies finding the DMHIs effective or partially effective in improving psychological outcomes. Although RCTs demonstrated high rates of adoption and adherence, these rates were lower in studies that used pre-post designs or implementation-effectiveness methodologies. Implementation and maintenance factors were seldom reported. When they were, the focus was primarily on recruitment sites and strategies. Based on these insights, we offer the following observations and recommendations for research going forward.

### Diversify the “ ” of DMHI

Our findings demonstrate a notable lack of diversity among the college students included in DMHI studies. The majority of DMHI studies took place on four-year university campuses with large student populations. Concentrating DMHI research in large universities neglects college students who could greatly benefit from these interventions. Community college students experience mental health issues more frequently than their peers in four-year universities and are less likely to use mental health services due to barriers like cost [53]. Additionally, many community college students are receptive to internet-delivered mental health interventions [54]. Establishing partnerships to test DMHI at smaller four-year schools and community colleges, like the STAND project (https://stand.ucla.edu/), could enhance the reach of DMHI to students with limited access to other treatments.

The samples in these studies were rather homogeneous, consisting mostly of female and non-Hispanic White students. This aligns with general trends in DMHI and traditional mental health treatment [55, 56], but is disappointing given the calls to expand recruitment to marginalized groups and cisgender men [57, 58]. Cisgender male students and students from traditionally marginalized groups are less likely to seek traditional therapy on college campuses due to stigma and concerns over culturally competent care [59, 60]. Attention to sexual and gender minorities was even rarer than racial and ethnic diversity, with only one-fifth of studies reporting on LGBTQ + students. LGBTQ + students in particular experience higher prevalence of mental health concerns relative to their heterosexual and cisgender peers [61]. To address the homogeneity of samples, active efforts must be made to include these students in DMHI initiatives. This includes tailoring intervention content, recruitment strategies [62], and human support to address fit with cultural/identity groups and personalization for each user [63].

### Move Beyond “Basic” Effectiveness Studies

Over two-thirds of the studies evaluated the effectiveness of specific DMHIs for mental health concerns, particularly anxiety and depression. Similar to past reviews [7••, 64], we found that most studies demonstrated that DMHIs were effective or partially effective at improving mental health, even when compared to waitlist or active controls. This consistent evidence underscores the effectiveness of DMHIs for college students in improving mental health concerns, especially for common issues such as depression and anxiety, and for promoting well-being and positive mental health.

Despite this, researchers frequently develop and test new DMHIs. The most studied DMHI was used in only 6% of studies, with most DMHI assessed in only one study. While it is important to assess these interventions, studies should also advance the field by evaluating DMHI with previously established effectiveness among college students in more diverse samples, and evaluating DMHI in the context of implementation on college campuses allowing consideration of practical implementation activities, long-term effectiveness, and sustainment. When novel DMHIs need to be evaluated, hybrid effectiveness-implementation designs can simultaneously provide information both about their impact as well as the necessary strategies and resources to support their implementation [12••, 65]. Studies should also be of sufficient size to consider moderator analyses such as demographic characteristics of students or their use patterns. Efforts to harmonize baseline measures and outcomes across studies could also support combined analyses and drive approaches to personalize care.

### Record and Publish Implementation and Maintenance Metrics

Implementation and maintenance were the least covered domains. Our review found that less than a fifth of studies mentioned implementing the DMHI within college campus structures and less than one-tenth reported on implementation activities. For research on DMHIs to translate to real-world contexts, proper attention needs to be given to these aspects to guide implementation attempts. This information is especially important for decisionmakers on college campuses, especially consideration of costs and maintenance [66, 67]. This work will require researchers to partner with organizations and administrators on campus and consider how to continue to collect data beyond the period of the traditional research study.

## Conclusions

We must acknowledge some limitations in our findings. First, we only used data presented within published articles; some research teams may have collected additional data relevant to RE-AIM that was not published. Second, our broad inclusion criteria led to significant heterogeneity in study methodologies, DMHI delivery formats, and psychological outcomes assessed. While this approach allowed us to provide a comprehensive overview of recent progress, it makes direct comparisons between studies difficult. Finally, we did not assess the quality and potential bias of the studies.

Our review offers several key strengths. As a systematic review, we could provide a comprehensive overview of the DMHI landscape for college students over the past five years. The RE-AIM framework was useful for identifying gaps in the literature to consider to improve DMHI dissemination and implementation.

Due to the growing concerns around student mental health, DMHIs offer an attractive option for college campuses to consider. However, the current research does not address questions that will be crucial to answer in order to integrate DMHI into practice. Therefore, although found effective in existing research, to realize their potential we need to improve DMHI and their delivery to reach and benefit a diverse range of students. This review highlights the advances seen in the past five years and demonstrates effectiveness in the studies undertaken thus far.

## Supplementary Information

Below is the link to the electronic supplementary material.

Supplementary file1 (XLSX 13 KB)

Supplementary file2 (XLSX 82 KB)

## Data Availability

No datasets were generated or analysed during the current study.
